# Decoding the multifaceted interventions between human sirtuin 2 and dynamic hepatitis B viral proteins to confirm their roles in HBV replication

**DOI:** 10.3389/fcimb.2023.1234903

**Published:** 2024-01-04

**Authors:** Zahra Zahid Piracha, Umar Saeed, Irfan Ellahi Piracha, Seneen Noor, Elyeen Noor

**Affiliations:** ^1^ Department of Medical Research, International Center of Medical Sciences Research (ICMSR), Islamabad, Pakistan; ^2^ Clinical and Biomedical Research Centre (CBRC) and Multidisciplinary Lab (MDL), Foundation University School of Health Sciences (FUSH), Foundation University, Islamabad, Pakistan; ^3^ Atta ur Rahman School of Applied Biosciences (ASAB), National University of Sciences and Technology (NUST), Islamabad, Pakistan

**Keywords:** HBV replication, sirtuins, sirtuin 2, Sirt2.1, Sirt2.2, Sirt2.5

## Abstract

The human sirtuin 2 gene (*SIRT2*) encodes a full-length Sirt2 protein (i.e., the Sirt2 isoform 1), which primarily functions as a cytoplasmic α-tubulin deacetylase, and which promotes the growth of hepatocellular carcinoma (HCC). Hepatitis B virus (HBV) replication itself, or HBV X (HBx) protein-mediated transcriptional transactivation, enhances Sirt2.1 expression; therefore, Sirt2.1 itself is capable of positively increasing HBV transcription and replication. Sirt2.1 is linked to liver fibrosis and epithelial-to-mesenchymal transition and, consequently, augments the risk of HCC. The Sirt2.1 protein enhances the HBV replication cycle by activating the AKT/glycogen synthase kinase 3 beta (GSK3β)/β-catenin pathway. It also activates the transcription of the viral enhancer I/HBx promoter (EnI/Xp) and enhancer II/HBc promoter (EnII/Cp) by targeting the transcription factor p53. The Sirt2 isoform 2 (Sirt2.2) is mainly localized in the cytoplasm, and the N-terminus is shorter by 37 amino acids than that of Sirt2.1. Despite the truncation of the N-terminal region, Sirt2.2 is still capable of enhancing HBV replication and activating the AKT/GSK3β/β-catenin signaling pathway. The Sirt2 isoform 5 (Sirt2.5) is primarily localized to the nucleus, it lacks a nuclear export signal (NES), and the catalytic domain (CD) is truncated. Upon HBV replication, expression of the Sirt2 isoforms is also enhanced, which further upregulates the HBV replication, and, therefore, supports the vicious cycle of viral replication and progression of the disease. Sirt2 diversely affects HBV replication such that its isoform 1 intensely augments HBV replication and isoform 2 (despite of the truncated N-terminal region) moderately enhances HBV replication. Isoform 5, on the other hand, tends to protect the cell (for smooth long-term continued viral replication) from HBV-induced extreme damage or death via a discrete set of regulatory mechanisms impeding viral mRNAs, the hepatitis B core/capsid protein (HBc), core particles, replicative intermediate (RI) DNAs, and covalently closed circular DNA (cccDNA) levels, and, consequently, limiting HBV replication. In contrast to Sirt2.1 and Sirt 2.2, the Sirt2.5-mediated HBV replication is independent of the AKT/GSK3β/β-catenin signaling cascade. Sirt2.5 is recruited more at cccDNA than the recruitment of Sirt2.1 onto the cccDNA. This recruitment causes the deposition of more histone lysine methyltransferases (HKMTs), including SETDB1, SUV39H1, EZH2, and PR-Set7, along with the respective corresponding transcriptional repressive markers such as H3K9me3, H3K27me3, and H4K20me1 onto the HBV cccDNA. In HBV-replicating cells, Sirt2.5 can also make complexes with PR-Set7 and SETDB1. In addition, Sirt2.5 has the ability to turn off transcription from cccDNA through epigenetic modification via either direct or indirect interaction with HKMTs.

## Introduction

Hepatitis B virus (HBV), an archetype within the Hepadnaviridae family, epitomizes a non-cytopathic hepatotrophic DNA virus, as expounded by Seeger and Mason in 2015. Its genome comprises partially double-stranded (DS), relaxed-circular (RC) DNA measuring 3.2 kb, which metamorphoses into covalently closed circular DNA (cccDNA) upon hepatocyte nucleus entry, as presented by Summers and Mason in 1982. This cccDNA assumes the form of a mini chromosomal structure, which is meticulously organized by a myriad of histones and non-histone viral and cellular proteins, as articulated by Saeed et al. (2021) and Saeed et al. (2018); Belloni et al. (2009); Levrero et al. (2009), and Ganem and Prince (2004). Notably, cccDNA serves as the transcriptional blueprint for diverse viral RNAs, including the 3.5-kb pre-genomic RNA (pgRNA), which is responsible for translating the core protein (HBc) and polymerase proteins. Additionally, the 2.4-kb and 2.1-kb subgenomic RNAs (sgRNAs) encode the large, middle, and small HBV surface proteins, with a 0.7-kb sgRNA encoding the HBV X (HBx) protein (Summers and Mason, 1982; Ganem and Prince (2004); Locarnini, 2004).

HBV infections can precipitate acute hepatitis, progressing to chronic stages, liver fibrosis, cirrhosis, and potentially hepatocellular carcinoma (HCC) (Seeger and Mason, 2015). Despite the existence of anti-HBV vaccines and limited therapeutic options, such as interferon alpha (IFN-α) and nucleos(t)ide analogs, a staggering 296 million individuals endure chronic hepatitis B (CHB) infection, as averred by Papatheodoridis et al. (2008); Testoni et al. (2017); Revill et al. (2019); Martinez et al. (2020), and the World Health Organization, 2017, and as corroborated by Saeed and Piracha (2023). Although anti-HBV regimens mitigate viral load, they fall short of effecting a cure due to the persistence of cccDNA. Remarkably, a lone copy of cccDNA holds the potential to advance the disease toward HCC. Existing therapeutics either falter in eradicating the virus or in suppressing cccDNA transcription, leaving the virus poised for reactivation post treatment (Guo and Guo, 2015; Testoni et al., 2017; Revill et al., 2019). Recognizing that the interaction between HBV and the host cells significantly shapes clinical outcomes, a strategic focus on the cellular factors contributing to viral replication emerges as a promising avenue for viral elimination.

Sirtuins (Sirts), which are integral members of the atypical class III histone deacetylase (HDAC) superfamily, encompass seven distinguished entities denoted as sirtuin1 to sirtuin7, according to de Ruijter et al. (2003). The mammalian Sirt family, homologous to the yeast silent information regulator 2 (Sir2 protein) and reliant on nicotinamide adenine dinucleotide (NAD)+ for its histone deacetylase activity, regulates diverse biological events such as stress responses, metabolism, and apoptosis, as elucidated by Finnin et al. (2001); North and Verdin (2004); Blander and Guarente (2004); Chalkiadaki and Guarente (2015), and Aventaggiato et al. (2021). The burgeoning literature increasingly reports on the interaction between sirtuins and viral infections, notably highlighted by the findings that sirtuin 1 (Sirt1) suppresses HBV transcription (Belloni et al., 2012). Correspondingly, Sirt1 and histone deacetylase 1 (HDAC1) collectively stymie cccDNA transcription in the absence of HBx (Belloni et al., 2009). Sirtuin 3 (Sirt3) and sirtuin 7 (Sirt7) employ epigenetic modifications to curtail HBV transcription and replication, as delineated by Ren et al. (2016) and Yu et al. (2021). Intriguingly, the oncogenic HBx protein interferes with mitochondrial Sirt4 expression, thereby downregulating it. Activation of Sirt4 induces cell apoptosis and enforces cell cycle arrest, notably at the G2/M checkpoint, which effectively impedes cell cycle progression in cancer cells. However, HBx counteracts the tumor-suppressive role of Sirt4, as reported by Huang et al., 2021. In the presence of sirtuin 5 (Sirt5), HBV replication is enhanced, although the molecular mechanisms underlying this phenomenon remain to be elucidated (Deng et al., 2017). Furthermore, sirtuin 6 (Sirt6) expression is modulated by HBV, yet the precise mechanisms governing virus-mediated modification remain elusive (Jenke et al., 2014; Yuan et al., 2021). HBV has the capacity to modify the expression levels of various SIRT proteins (Pereira et al., 2018; Piracha et al., 2018; Piracha et al., 2020; Kong et al., 2021).

The spotlight on sirtuin 2 (Sirt2) in viral infection has intensified in recent years. Recognized as a pivotal target for dengue viral infection (Wan et al., 2021), Sirt2 has been implicated in augmenting HBV replication (Cheng et al., 2018). Remarkably, an inhibitor of Sirt2 has demonstrated anti-HBV effects, as validated by Yu et al., 2018. Despite these advancements, further investigation is required to meticulously unravel the underlying mechanisms of action. Sirt2, a pivotal regulator of acetylation, phosphorylation, and substrate localization, plays a crucial role in governing these processes.

## Hepatitis B virus-induced molecular conformations on human sirtuin 2

The effects of HBV on Sirt2 have been extensively investigated by several groups (Wu et al., 2022). HBV replication is upregulating the endogenous expression of Sirt2 mRNA. The expression of different mRNAs of all three Sirt2 isoforms was higher in HBV-replicating cells than in the mock, as confirmed by RNase protection assays (RPAs). In RPAs, the length of protected mRNAs of Sirt2.1, Sirt2.2, and Sirt2.5 is 350 nucleotides (nt), 290 nt, and 180 nt, respectively. Furthermore, HBV replication also increases the protein expression of all three isoforms of Sirt2. Likewise, Sirt2 protein expression is low when HBV was impaired by either replication-deficient mutants (e.g., TP-Y65A and RT-YMHA) (Kim et al., 2004) or by RT inhibitors (e.g., lamivudine and entecavir). Immunoblot analysis revealed that Sirt2 proteins are expressed in both tumor and adjacent non-tumor liver tissues; however, HBV-associated HCC-biopsied tumor liver tissue expressed increased levels of Sirt2 as compared with adjacent non-tumor tissue (Chen et al., 2013). Furthermore, there are higher levels of HBc expression in the biopsied tumor than in the adjacent non-tumor liver tissues, which emphasizes the fact that Sirt2 and HBV together play a role in HBV replication. In the same way, Sirt2 mRNA and protein levels were found to be increased in peripheral blood mononuclear cells (PBMCs) in CHB patients, as compared with healthy individuals (Wu et al., 2022). Cheng et al. reported that the overexpression of Sirt2 mRNAs and its proteins is induced by HBx, as HBx is capable of activating the promoters of Sirt2 in HBV-positive hepatoma cells (Chen et al., 2013). Also, HBx is capable of transforming HBV to HCC (Cheng et al., 2018).

## Sirtuin 2-induced molecular interventions in hepatitis B virus replication and its life cycle

### Contemplating the molecular mechanisms of Sirt2.1-mediated enhanced HBV replication in an HBx-independent manner

**Figure 1:**
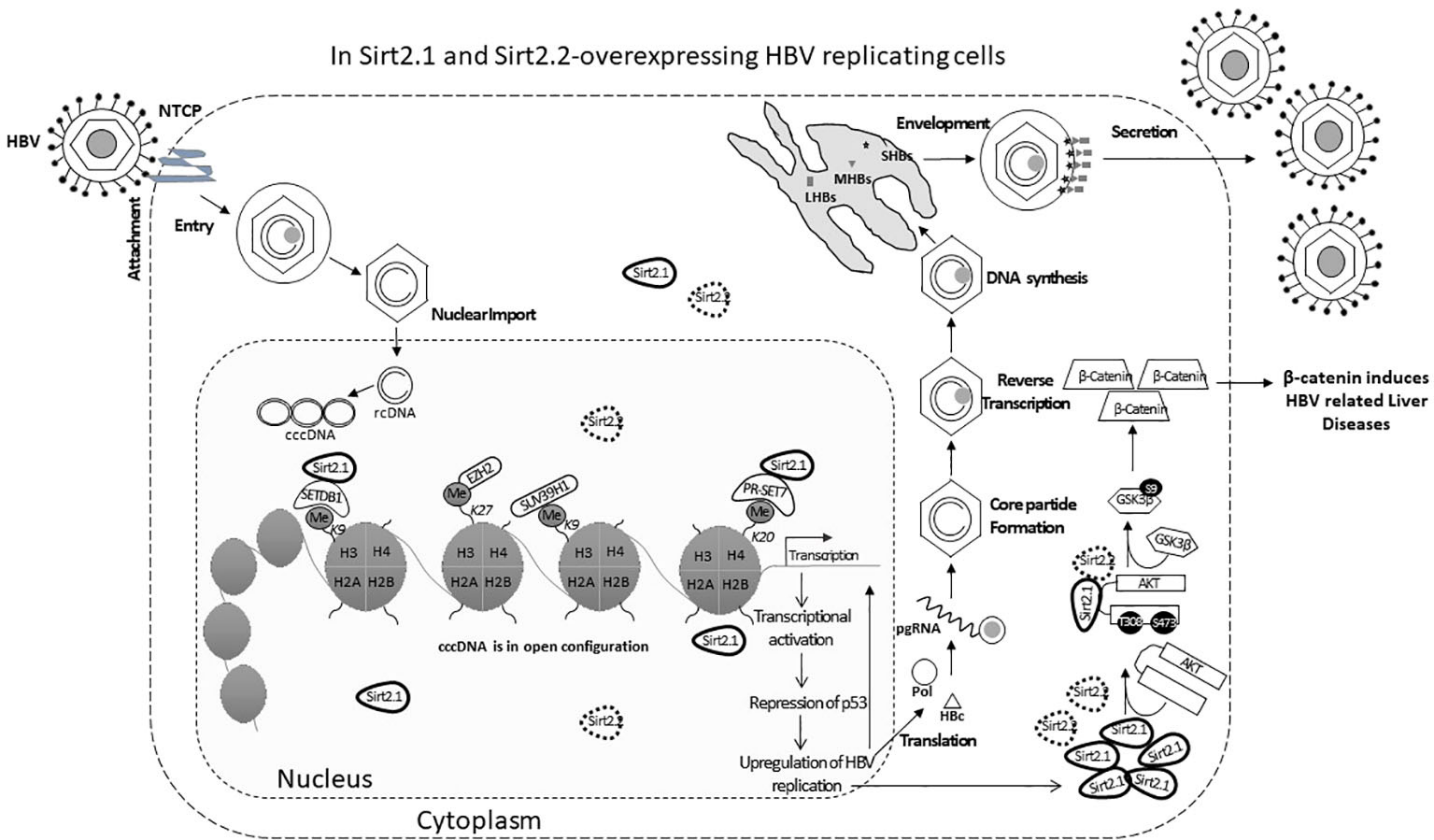
The Sirt2 isoforms 1 and 2 enhance HBV replication in an HBx-independent manner via the AKT/GSK-3β/β-catenin signaling pathway. In Sirt2.1- and 2.2-overexpressing cells the HBV core protein, HBV transcription levels, HBV mRNAs, core particle levels, and HBV DNA levels are significantly enhanced. Sir2.1 and 2.2 enhanced HBV replication in an HBx-independent manner through the AKT/GSK-3β/β-catenin signaling cascade, thus supporting HBV propagation and virion egression.

Sirt2.1 is a full-length protein transcribed from the *SIRT2* gene. The structure of Sirt2.1 consists of a large Rossmann fold domain (involving residues 55–91, 146–186, and 241–356) and a small zinc-binding domain (involving residues 92–145 and 187–240) (Yamagata et al., 2014). Sirt2.1 overexpression enhanced all enhancers and promoter activity of HBV [enhancer 1/X promoter (EnhI/Xp), enhancer 2/Core promoter (EnhII/Cp), preS1p, and preS2p]. Furthermore, HBV pgRNA and subgenomic small RNA (smRNA) levels were also enhanced (**Figure 1**). Sirt2.1 overexpression is reported to increase the protein levels of HBs [i.e., large HBs (LHBs), medium HBs (MHBs), and small HBs (SHBs)], and HBc protein levels (**Figure 1**). Also, the formation of a core particle and HBV DNA synthesis is amplified upon Sirt2.1 overexpression (**Figure 1**) (Wu et al., 2022). The Sirt2.1 overexpression also promoted secretion levels of HBsAg and HBeAg (**Figure 1**) (Wu et al., 2022). The mechanistic effect of Sirt2 on the secretion of HBsAg and HBeAg was not studied in detail. Examining the potential interactions between Sirt2, HBsAg, and HBeAg and their subsequent effects on viral propagation or HBV virion secretion are important steps toward understanding Sirt2-induced viral replication. Inhibiting Sirt2.1 expression, either by knocking down, silencing, inhibiting, or dominant negatively mutating Sirt2.1, can decrease HBV replication (Wu et al., 2022; Cheng et al., 2018).

The underlying mechanism of Sirt2-induced HBV replication is still not clear. One study reported that Sirt2 facilitates the epithelial-to-mesenchymal transition in HCC via the AKT/GSKβ/β-catenin signaling cascade (Chen et al., 2013). This signaling cascade is also involved in HBV replication since Sirt2.1, as a deacetylase, interacts with AKT and helps in the full activation of AKT (**Figure 1**) (Ramakrishnan et al., 2014). This Sirt2–AKT/GSKβ/β-catenin signaling cascade-mediated HBV replication is not dependent on HBx. A recent study speculated that the effects of Sirt2.1 on the replication of HBV might be dependent on HBV enhancers and promoters. Sirt2.1 aids HBV replication by repressing the binding of p53 to HBV enhancers and promoters (EnI/Xp and EnII/Cp) (Wu et al., 2022). Sirt2.1 is also recruited to cccDNA where it interacts less with histone lysine methyltransferases (HKMTs), such as PR-Set7 and the SET domain bifurcated histone lysine methyltransferase 1 (SETDB1), on HBV cccDNA, which in turn causes less deposition of the transcription suppressive markers onto cccDNA and switches on transcription from the cccDNA (**Figure 1**).

## Molecular interventions of Sirt2.2 to hijack the cellular pathways for enhanced HBV propagation

**Figure 2:**
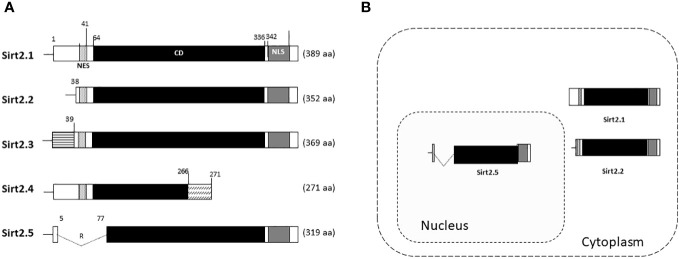
Schematic diagram of Sirt2 isoforms and residues are numbered according to the full-length Sirt2 isoform 1. **(A)** The Sirt2 isoform 1 has NES, CD, and NLS domains and is composed of 389 amino acids. The Sirt2 isoform 2 has common NES, CD, and NLS domains, while the N-terminal region is truncated and consists of 352 amino acids. The isoforms 3 and 4 consist of 369 and 271 amino acids, respectively. For Sirt2.5, the NES and/or N-terminal-truncated catalytic domain consists of 319 amino acids. **(B)** In contrast to the Sirt2 isoform 1 and/or 2, isoform 5 is mainly localized in the nucleus.

The effects of Sirt2.2 on HBV replication were not extensively investigated in regard to viral replication. To our knowledge, one single study was the first to probe into the effects of Sirt2.2 on HBV replication (Piracha et al., 2020). In contrast to Sirt2.1, Sirt2.2 lacks the first 37 amino acids from the N-terminal (**Figure 2A**) (North and Verdin, 2007). These amino acids might be critical for HBV replication, because the presence of these amino acids in Sirt2.1 has been shown to have a distinctive role in the remarkable augmentation of HBV replication, as compared with Sirt2.2. It might be speculated that the absence of these amino acids in Sirt2.2 inhibits its ability toward binding to the cccDNA for transcription. It is interesting to mention here that, despite the truncated N-terminal domain, Sirt 2.2 can unequivocally augment HBV replication by several folds via enhanced core protein levels, HBV transcripts (pgRNA, smRNAs), core particle formation, elevation of the AKT/GSKβ/β-catenin signaling cascade, and, consequently, augmented HBV replication levels. How Sirt2.1 plays a more critical role in HBV replication as compared with Sirt2.2 is still debatable and needs further investigation. However, based on the current knowledge, it has been speculated that the difference in inducing the HBV replication is due to the fact that Sirt2.1 is recruited more toward cccDNA and is capable of inducing the open conformation of the cccDNA for further improved transcription. Sirt2.2 can easily shuttle between the cytoplasm and the nucleus. The first 37 amino acids are critical for binding to chromatin. On Sirt2.2 knockdown the formation of HBc, synthesis of the core particle, transcription of HBV mRNAs, and HBV DNA synthesis were significantly reduced. This reduction affirmed the potential roles of Sirt2.2 in HBV replication in an HBx-independent manner. Since the main cellular localization of Sirt2.2 is the same as that of Sirt2.1, that is, the cytoplasm (**Figure 2B**) (Rack et al., 2014), it can be speculated that they may have overlapping effects in HBV replication. This localization of Sirt2.2 was not altered when HBV is replicating in the cells. Sirt2.2 also upregulated HBV replication through the AKT/GSKβ/β-catenin signaling cascade. The effects of Sirt2.2 on HBsAg levels has not been investigated yet. It would be more interesting to examine the further effects of Sirt2.2 on HBsAg and HBeAg levels and their subsequent effects on the occurrence of HCC among HBV patients.

## Sirt2.5 thwarted HBV replication without the AKT/GSKβ/β-catenin signaling pathway in an HBx-independent manner

In Sirt2.5, as compared with Sirt2.1, the nuclear export signal (NES) and part of the catalytic domain (CD) are removed as a result of a splicing event in which 6 to 76 amino acids are spliced out and replaced by amino acid arginine (CGT) (**Figure 2A**) (Rack et al., 2014). As a result, Sirt2.5 lacks deacetylase activity for most of the known Sirt2 substrates (Rack et al., 2014). It is also established that the NES and CD of Sirt2 is important in deacetylase activity (Rack et al., 2014). Sirt2.5 overexpression decreased the transcriptional activity from all viral enhancers and promoters. Accordingly, the levels of HBV pgRNA and subgenomic smRNAs also decreased upon overexpression of Sirt2.5. Similarly, the levels of HBc, core particle formation, and HBV DNA synthesis decreased drastically when Sirt2.5 is overexpressed in hepatoma cells. When Sirt2.5 expression is inhibited, HBV replication is enhanced.

**Figure 3:**
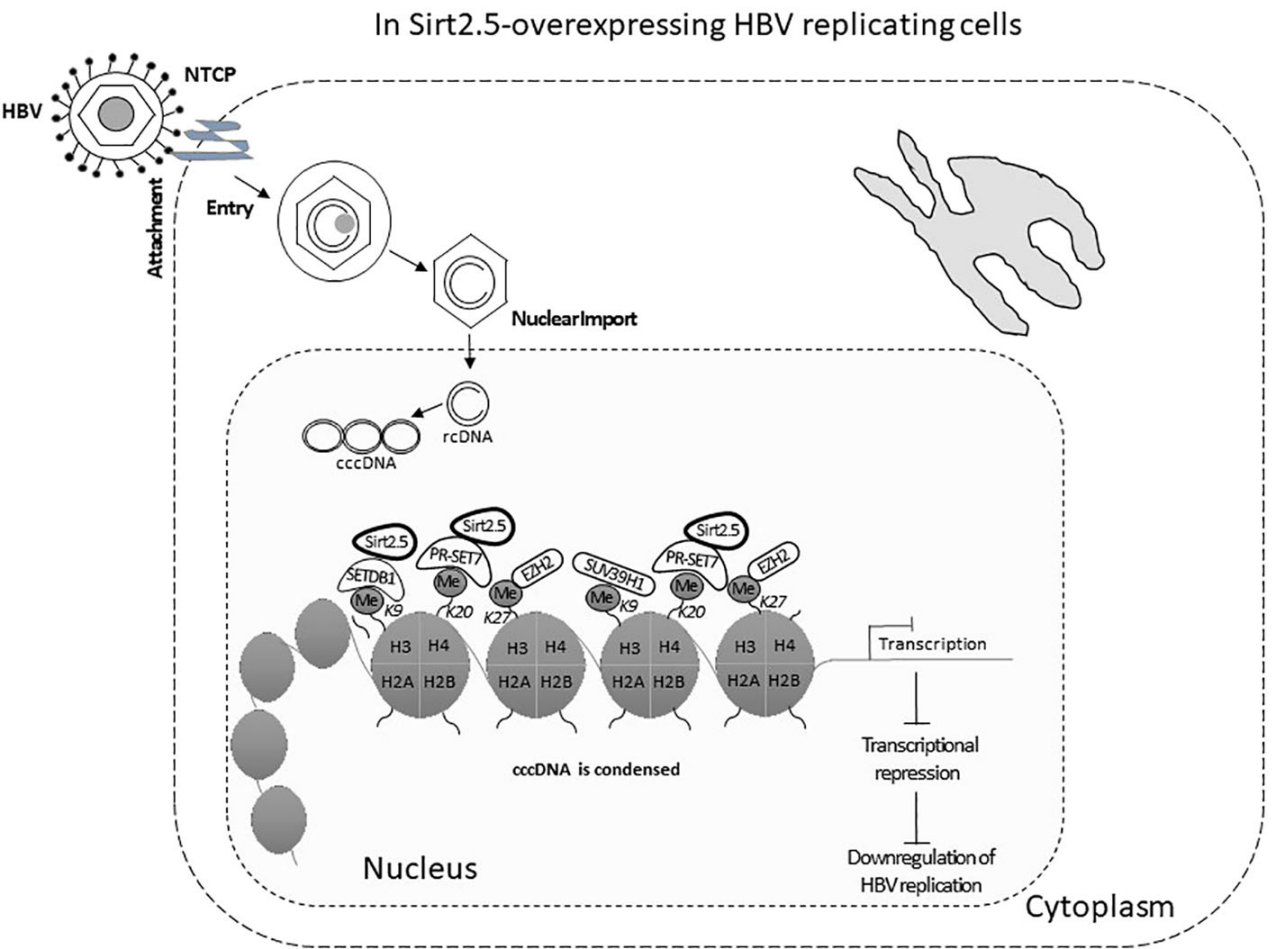
The Sirt2.5 is critically involved in reducing HBV replication. The catalytically inactive nuclear isoform 5 of Sirt2 antagonizes the effects of isoforms 1 and 2 on HBV replication by inhibiting cccDNA production, viral mRNAs, and, consequently, lowering the synthesis of replicative intermediate DNA. Sirt2.5 more firmly binds to cccDNA, as compared to Sirt2.1, and facilitates epigenetic modification by improving the deposition of transcriptional repressive markers like histone lysine methyl transferases that repressed HBV transcription.

Sirt2.5 does not affect HBV replication via AKT, as the preferred localization for Sirt2.5 is the nucleus (**Figure 2B**) and the Sirt2.5–AKT interaction weakens upon HBV replication. So, the Sirt2.5-mediated downregulation of HBV does not involve the AKT/GSKβ/β-catenin signaling cascade or HBx. Since the main localization of Sirt2.5 is in the nucleus, it is primarily recruited to cccDNA chromatin structure (**Figure 3**). In Sirt2.5-overexpressing HBV-infected cells, different HKMTs, such as the suppressor of variegation 3–9 homolog 1 (SUV39H1), SETDB1, an enhancer of zeste homolog 2 (EZH2), and PR-Set7, are also recruited to cccDNA and cause the deposition of transcription suppressive markers such as trimethyl-H3K9 (H3K9me3), monomethyl-H4K20 (H4K20me1), and trimethyl-H3K27 (H3K27me3). Sirt2.5 interacts with PR-Set7 and SETDB1 on HBV cccDNA and turns off transcription from cccDNA (**Figure 3**).

## Multifaceted sirtuins (isoforms 1 and 5) affect the HBV life cycle

HBV can increase the expression level of *SIRT2* and its alternatively spliced transcript variants Sirt2.1 and Sirt2.5. However, the two isoforms show opposite effects on HBV replication. As mentioned above, overexpression of Sirt2.1 augments HBV replication at different stages, from formation of HBV cccDNA to the synthesis of RNA and DNA (Wu et al., 2022). These effects are independent of HBx. The different subcellular localizations of the SIRT2 protein (Sirt2.1 in the cytoplasm and Sirt2.5 in the nucleus) may be a distinguishing feature of their multifunctional roles in the HBV life cycle (**Figure 2B**) (Rack et al., 2014).

Northern blotting, luciferase, and chromatin immunoprecipitation assays revealed that overexpression of Sirt2.1 in HepG2 and Huh7 cells caused transcriptional activation and augmented the levels of several HBV RNAs. These changes resulted in enhanced levels of HBc, core particle formation, and DNA synthesis. However, overexpression of Sirt2.5 in HepG2 and Huh 7cells showed the opposite effects.

Knockdown of *SIRT2* in cultured liver cell lines (HepG2 or Huh7) or in HBV infection systems (i.e., HepG2-NTCP or primary human hepatoma) abrogated the levels of cccDNA, pgRNA, sgRNAs, and HBc, core particle assembly, and synthesis of HBV DNA (Wu et al., 2022). ELISA specified that SIRT2 depletion in HBV infection system also depleted the levels of HBsAg and HBeAg in the supernatant (Wu et al., 2022). RNA decay assays and nascent RNA capture assays performed in HepG2-NTCP cells revealed that overexpression or silencing of *SIRT2* has no effect on the half-life of pgRNA or total HBV RNAs. This indicated that SIRT2 does not affect the degradation rate of HBV RNA and the alteration of levels of total HBV RNAs are instigated by the effect of SIRT2 on HBV RNA production rather than on HBV RNA stability (Wu et al., 2022). Likewise, treatment of the cell lines with a SIRT2 inhibitor such as AGK2 {2-cyano-3-[5-(2,5-dichlorophenyl)-2-furanyl]-N-5-quinolinyl-2-propenamide} (Outeiro et al., 2007) reduced HBV replication significantly (Yu et al., 2018). The effects of AGK2 on HBV replication were also determined in HBV transgenic mice (Yu et al., 2018). Serum samples and liver tissues were studied in detail. The data from mice serum alanine transaminase (ALT) and aspartate transaminase (AST) showed that AGK2 is not toxic to liver cells *in vivo.* The HBV DNA level in mice serum was analyzed by using real-time PCR. The level of HBV DNA in the AGK2-treated group was diminished. Subsequently, HBV DNA levels and serum levels of HBsAg/HBeAg also lessened on AGK2 treatment. The levels of HBV pgRNA and total RNAs, extracted from mouse liver tissues, also decreased in the drug-treated group, as compared with control mice group (Yu et al., 2018). These findings strengthen the evidence that Sirt2 is important for HBV replication (Yu et al., 2018; Wu et al., 2022).

As mentioned above, overexpression of Sirt2.1 helps transcription and replication of HBV by suppressing the binding of p53 to HBV EnI/Xp and EnII/Cp (Wu et al., 2022). Another study stated that Sirt2.1 facilitated the AKT/GSK-3β/β-catenin pathway and its role in HBV replication. Furthermore, this role is independent of HBx. However, Sirt2.1 itself is upregulated by HBx and this upregulation of Sirt2 helps in the progression of HBV-induced HCC or hepatocarcinogenesis (Cheng et al., 2018). Hence, Sirt2.1 augments HBV replication. Overexpression of Sirt2.5 has the opposite effect and this effect does not involve HBx or the AKT/GSK-3β/β-catenin pathway. Overexpression of Sirt2.5 in HBV-replicating cells reduced the recruitment of RNA polymerase II, and acetylated H3 onto cccDNA, indicating transcriptional repression. Different HKMTs, such as PR-Set7, SETDB1, EZH2, and SUV39H1, recruited more to cccDNA and to the deposition of the repressive markers of transcription, such as H3K9me3, H3K27me3, and H4K20me. The recruitment of SET1A, which methylates H3K4 to trigger transcription (Alarcon et al., 2016), is also reduced in Sirt2.5-overexpressing HBV-replicating cells. These changes might trigger the coiling of cccDNA from the open to the closed conformation, thereby suppressing HBV transcription, and in turn HBV DNA replication (**Figure 3**).

## Conclusions and perspectives

HCC is the most common and malignant type of cancer and is the third most common cause of cancer-related deaths globally (Bruix et al., 2014). Almost 750,000 new HCC cases are reported each year and the number is still increasing (Jemal et al., 2011). Several proteins and pathways are involved in HCC progression and metastasis; one of the proteins studied is Sirt2, as it facilitates epithelial-to-mesenchymal transition and supports the metastasis of HCC via the AKT/GSK-3β/β-catenin pathway (Chen et al., 2013). High Sirt2 levels are also linked with advanced tumor stage and a low rate of patient survival (Chen et al., 2013). CHB infection is a major cause of HCC and higher levels of HBV replication attributed to the development of HCC (Kawanaka et al., 2014). Therefore, one therapeutic approach to improve the cure rate is to target host factors contributing to HBV replication.

Viruses interact with different cellular host factors during different stages of their life cycle (Ligat et al., 2021). They create the environment advantageous to their replication by enhancing or degrading the expression of different host genes (Duggal and Emerman, 2012; Nakagawa and Makino, 2021). The chemistry between the virus and cellular host factors regulates the pathogenicity and infectivity of the virus (Wißing et al., 2021). Sirt2 is one such cellular host factor carrying the function of an α-tubulin deacetylase (North and Verdin, 2004). AGK2, the potent inhibitor of Sirt2 enzymatic activity, could significantly decrease HBV transcription and replication, signifying the fact that the importance of Sirt2 in regulating the HBV lifecycle may depend on its deacetylase activity (Yu et al., 2018). Different isoforms have different effects on HBV replication. It is surprising to know that a single gene of *SIRT2* that makes primary (Sirt2.1) and alternatively spliced transcripts (Sirt2.5) can exert completely opposite effects on HBV replication. We can assume that highly active HBV replication brought about by overexpression of Sirt2.1 and Sirt2.2 is somehow repressed to some extent by the increased Sirt2.5 expression. This could be a possible mechanism of self-regulation that modulates viral replication in order to avoid damage to the host cell and virus. It is important to find out the different epigenetic regulations of cccDNA for developing epigenetic therapy for shutting down HBV in infected hepatocytes. A complete understanding of the balance between the different readers (i.e., proteins that identify epigenetic regulations to bring change in gene expression) and erasers (i.e., enzymes that remove epigenetic marks) can help to silence the cccDNA. Sirt2.1 has shown supportive roles toward HBV replication such that the cellular protein enhanced viral HBV DNA levels and HBsAg, which in turn have the potential to worsen CHB leading toward HCC. The identification and characterization of specific Sirt2 inhibitors may lead to the development of new treatment options for CHB. Alternatively, the knockdown of *SIRT2* to inhibit HBV replication or overexpression of Sirt2.5 could also be explored as a therapeutic option. Right now, no therapeutic strategies are utilizing Sirt2 as molecular targets. Considering the potential importance of Sirt2 in HBV infection, a better understanding of Sirt2-specific molecules, which can then be used for *in vivo* studies, could be of great benefit for finding the cure for CHB. Still, much research is needed to understand the interaction between Sirt2 and host cells and ultimately the effects on HBV replication to allow for the development of novel antiviral therapeutic agents.

## Author contributions

ZP conducted the study, wrote the manuscript, and was the principal investigator (PI) of the study. US assisted ZP to revise the study, assisted in critical revisions of the manuscript, and was Co-PI of the study. IP, SN, and EN provided technical assistance to ZP and US for the study. IP facilitated the interpretation and contributed to the discussion, data curation, visualization, methodology, and work design, and reviewing and editing. SN facilitated manuscript writing, contributed to analysis/interpretation, critical review, designing/planning, manuscript proofreading, data curation, validation, and investigation. EN contributed to the methodology, validation, manuscript writing review of second draft, material analysis, final editing, project administration, and data curation. All authors contributed to the article and approved the submitted version.
